# Triboelectric Hydrogen Gas Sensor with Pd Functionalized Surface

**DOI:** 10.3390/nano6100186

**Published:** 2016-10-14

**Authors:** Sung-Ho Shin, Yang Hyeog Kwon, Young-Hwan Kim, Joo-Yun Jung, Junghyo Nah

**Affiliations:** 1 Department of Electrical Engineering, Chungnam National University, Daejeon 34134, Korea; shinsh@cnu.ac.kr (S.-H.S.); yhkwon@cnu.ac.kr (Y.H.K.); kyhwan@cnu.ac.kr (Y.-H.K.); 2 Department of Nano Manufacturing Technology, Korea Institute of Machinery and Materials, Daejeon 34103, Korea

**Keywords:** self-powered sensor, H_2_ detection, palladium, gas sensor, triboelectricity

## Abstract

Palladium (Pd)-based hydrogen (H_2_) gas sensors have been widely investigated thanks to its fast reaction and high sensitivity to hydrogen. Various sensing mechanisms have been adopted for H_2_ gas sensors; however, all the sensors must be powered through an external battery. We report here an H_2_ gas sensor that can detect H_2_ by measuring the output voltages generated during contact electrification between two friction surfaces. When the H_2_ sensor, composed of Pd-coated ITO (indium tin oxide) and PET (polyethylene Terephthalate) film, is exposed to H_2_, its output voltage is varied in proportion to H_2_ concentration because the work function (WF) of Pd-coated surface changes, altering triboelectric charging behavior. Specifically, the output voltage of the sensor is gradually increased as exposing H_2_ concentration increases. Reproducible and sensitive sensor response was observed up 1% H_2_ exposure. The approach introduced here can easily be adopted to development of triboelectric gas sensors detecting other gas species.

## 1. Introduction

Hydrogen (H_2_) has been claimed to be an alternative to replace fossil fuels [1,2,3,4]. Since H_2_ is flammable, explosive, colorless, and odorless gas, precise detection of its concentration is important for prevention of potential accidents and its safe use [5]. Thus, many H_2_ sensors, exploiting different sensing mechanisms, have been introduced to date [6,7,8,9]. Among them, palladium (Pd)-based H_2_ sensor is one of the most widely used H_2_ sensors due to its fast reaction to H_2_, high sensitivity, and selectivity [10,11,12,13,14,15,16,17,18,19]. When Pd is exposed to H_2_ molecules, Pd is reacted with H_2_ by absorbing H_2_ molecules inside its lattice, expanding the volume: Pd is transformed as either α-phase PdH*_x_* or β-phase PdH*_x_* depending on exposed H_2_ concentrations. Consequently, the electrical and optical properties of Pd are changed as it is exposed to H_2_. Using this mechanism, several H_2_ sensors have been reported. For instance, H_2_ sensors, utilizing nanogap structures [10,11], surface acoustic wave (SAW) [13,14], Schottky diodes structure [15,16], and surface plasmon effect [17,18], have been reported. Recently, triboelectric energy harvesting has gained much attention as a route to supply necessary power to operate electronic devices [20,21,22,23,24,25,26]. Further, few attempts have been made to develop chemical sensors utilizing triboelectric effect [27,28]. Specifically, detection is usually made by measuring variable triboelectric output as a result of friction surface reaction with target gas molecules [27]. However, only limited chemical species have been probed so far and further investigation is necessary to expand its application.

Here, we report a self-powered H_2_ gas sensor by adopting triboelectric effects. When two dielectric surfaces on metal electrode are periodically contacted and released, each surface is electrified due to charge separation between two surfaces. The surface that gained electrons is charged negatively, while the other surface that lost electrons is positively charged, depending on their difference in electron affinity. These surface charges electrostatically induce counter charges on the facing metal electrode, developing the potential difference between two metal electrodes. Therefore, when the two electrodes are connected through an external load, it drives the electrons to flow between them. In this work, based on this triboelectric generation mechanism, we report a self-powered H_2_ gas sensor, demonstrating reliable sensing performance from 0% to 1% H_2_ exposures.

## 2. Results and Discussion

### 2.1. Preperation of Self-Powered H_2_ Gas Sensor

**Figure 1:**
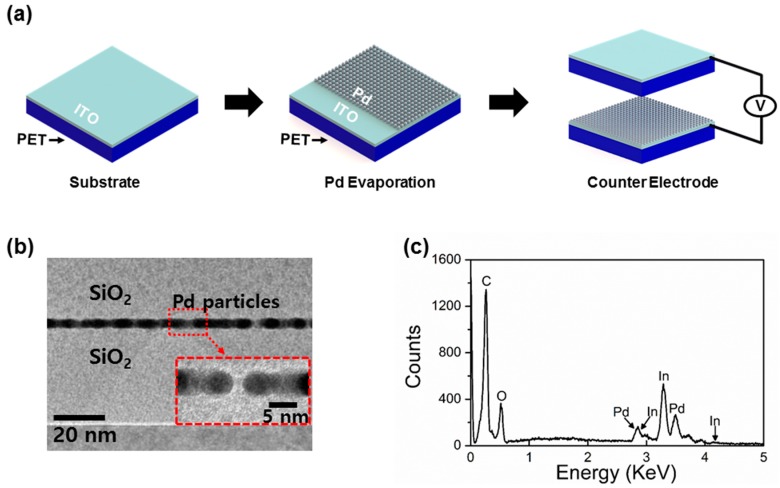
(**a**) Schematic representations of self-powered H_2_ gas sensor fabrication. First, a 10 nm-thick Pd is thermally evaporated on an indium tin oxide (ITO) surface. The courter surface consists of the polyethylene Terephthalate (PET) and ITO layer. Two contacting surfaces, PET and ITO surfaces, were periodically pressed and released during measurement. Triboelectric output voltages were measured while exposing the devices at different concentrations of H_2_ gas; (**b**) transmission electron microscopy (TEM) shows the thickness of evaporated Pd layer, ~10 nm; (**c**) energy-dispersive X-ray spectroscopy (EDS) of Pd coated ITO electrode. It clearly indicates the contents of Pd deposited on an ITO/PET substrate.

The schematic representation of the self-powered Pd H_2_ gas sensor is shown in Figure 1a. Two indium tin oxide (ITO) (130 nm)/polyethylene Terephthalate (PET) (130 μm) substrates (dimension: 1 cm × 1 cm × 130.13 μm) are first cleaned with IPA acetone and DI water, respectively. On one substrate, a 10 nm-thick Pd is thermally evaporated on the ITO side of the substrate as an active H_2_ sensing layer. The other substrate is used as the contacting substrate without any chemical treatment or coating, where the PET side of the substrate is employed as a counter contact surface. Lastly, ITO electrodes of the two substrates are connected through an external circuit. Figure 1b shows the cross sectional transmission electron microscopy (TEM) of the evaporated Pd. The Pd layer id co-deposited on a Si/SiO_2_ substrate in order to measure the thickness of the Pd layer. It can be noticed that ~10 nm evaporated Pd is deposited on the substrate. Next, energy-dispersive X-ray spectroscopy (EDS) was also examined to verify the evaporated Pd layer on the ITO surface (Figure 1c).

### 2.2. Self-Powered H_2_ Gas Sensor Test Set up

**Figure 2:**
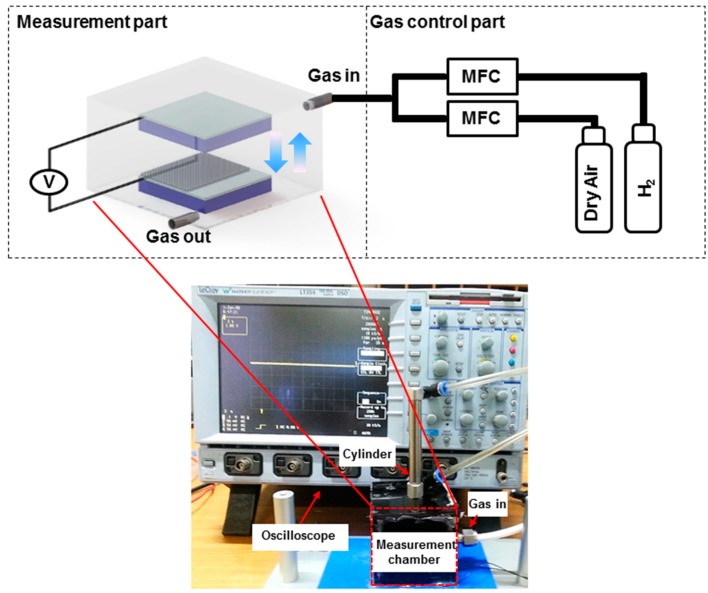
H_2_ gas sensor test setup consisting of gas control part and measurement part. A specific concentration of H_2_ is precisely controlled by mixing dry air and H_2_ at different ratio and the mixed gas is then introduced into the measurement chamber with a custom-made pushing machine installed inside. The output signals from the sensor were measured using the data acquisition instrument.

For self-powered H_2_ gas sensor measurement, the test setup shown in Figure 2 was prepared. The test setup is composed of two parts: gas flow control unit, exposing gas concentration using mass flow control units (MFCs) by introducing 5% H_2_ and dry air at specific ratios, and measurement unit, measuring voltage between two electrodes by periodic contact. Experimental details of gas exposure are described in the Materials and Methods section. A specific concentration of H_2_, prepared in the gas control part, flowed into an enclosed gas chamber (length × width × height: 6 cm × 6 cm × 6 cm), within which the custom-built pushing machine is installed. Two surfaces of the H_2_ sensor were mounted on the pushing machine and were contacted periodically during pressing and releasing motions.

### 2.3. Mechanism and Output Performance of Self-Powered H_2_ Gas Sensor

**Figure 3:**
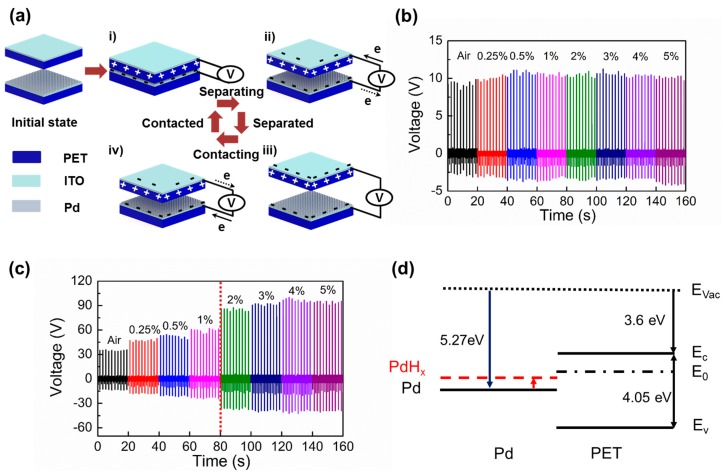
(**a**) Triboelectric generation mechanism. (initial state) There is no charge on both surfaces. (i) contacted state: charge separation between the two surfaces by contact electrification; (ii) releasing state: differently charged contact surface induces charge flows between the top and bottom electrodes; (iii) separated state: charge neutral state is reached; (iv) pressing state: electrical equilibrium state breaks again and induced charges flow in opposite direction; (**b**) Triboelectric output voltages from non Pd-functionalized ITO and PET contact pair. There is no output voltage change since Pd does not react with H_2_ gas; (**c**) Triboelectric output voltage of the H_2_ gas sensor with a Pd-functionalized ITO and PET contact pair in varying H_2_ concentrations. The output voltage sublinearly increases until H_2_ concentration reaches 1%. The output voltages are saturated above 3% H_2_ concentration; (**d**) Energy band diagrams of contact surfaces. Work function (WF) of Pd moves close to donor surface states (E_0_) of PET as PdH*_x_* is formed, increasing surface charge exchange.

Figure 3a shows the sensing mechanism of a self-powered H_2_ gas sensor, driven by triboelectric induction mechanism [29]. At the initial state, both Pd and PET surfaces have no surface charges. As the two surfaces are compressed, the electrons from donor interface states of the PET surface move toward Pd-functionalized ITO electrode through an external circuit, negatively charging the Pd-functionalized surface and positively charging the PET surface (Figure 3(ai)) [20]. As the two surfaces start to become separated, the surface charges on both contacting surfaces induce counter charges on the facing electrodes, developing the potential difference between the top and bottom electrode (Figure 3(aii)). At the fully separated state, charge equilibrium state is reached between the contacting surfaces and facing electrodes (Figure 3(aiii)). Lastly, as the two surfaces make contact again, induced charges on the electrodes disappear again due to a break of the charge neutral state (Figure 3(aiv)). To demonstrate the roles of Pd-functionalization, output voltages of the sensor without Pd-functionalized surface were first investigated (Figure 3b). When the non Pd-functionalized ITO and PET surfaces are contacted, it exhibits constant output voltage for varying H_2_ gas concentration from 0% to 5%. Since both PET and ITO do not react with H_2_ molecules, the output voltage signals were solely determined by the initial triboelectric sequence difference between them. Next, the output signals between Pd-functionalized ITO surface and PET were examined as a function of different exposing H_2_ concentrations. Differently from the non-functionalized device, it can be clearly noticed that the output voltages of the sensor are increased in proportion to H_2_ exposure (Figure 3c). The sensor output is more linearly increased up to 1% H_2_ exposure. However, the sensor response is rather saturated when the H_2_ concentration exceeds 2%. The observed behavior is consistent with other Pd-based hydrogen gas sensor [11,14]. Thus, Pd-functionalization plays a key role in triboelectric H_2_ detection. In Figure 3d, the energy band structure of the sensor is drawn to explain the increase of triboelectric output voltage with exposure to H_2_. The work function (WF) of Pd is ~5.2 eV and thus its Fermi level is located slightly above the midgap of PET [30]. As a result, when the two surfaces are contacted, carriers in valence and conduction bands of PET cannot be responsible for observed triboelectric charge exchange between the two surfaces. Therefore, the surface states must play an important role in contact electrifications between the two materials. Here, when the Pd-coated ITO surface is contact-electrified against the PET surface, Pd is negatively charged while PET is positively charged. This indicates that there must be donor states (0.55 and 0.85 eV below conduction band) near the midgap of PET, located slightly above the Fermi level of Pd [31]. As Pd is being exposed to H_2_, H_2_ molecules are dissociated and absorbed in the Pd lattice, forming PdH*_x_* (palladium hydride). When PdH*_x_* is formed, its WF becomes smaller than that of Pd [32]. Consequently, as the WF of PdH*_x_* moves more close to donor states in PET, the charge exchange between two materials can be more effective, which enhances surface charge exchange and leads to increase in triboelectric output voltage of the sensor. Therefore, triboelectric output voltage will be continuously increased until H_2_ concentration reaches a certain level.

**Figure 4:**
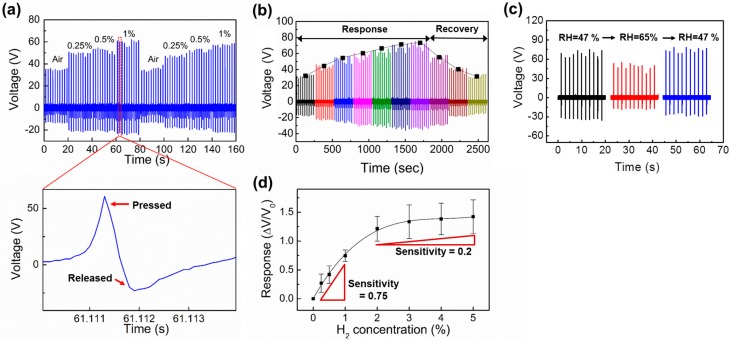
(**a**) Reproducible sensor responses. Similar outputs are repeatedly measured when the sensor is repeatedly exposed to H_2_; (**b**) Sensor response and recovery time under 1% hydrogen exposure; (**c**) Humidity effect on sensor output performance; (**d**) Sensor responses (*ΔV/V_0_*) under exposure to different concentrations of H_2_.

To verify reproducible output performance of the H_2_ sensor, the device was measured while repeatedly being exposed to different concentrations of H_2_ (up to 1% H_2_) (Figure 4a). Similar output voltages were measured during the repeated measurement. The magnified output signals in Figure 4a show alternating peaks due to the triboelectric induction mechanism described in Figure 3a. Next, sensor response and recovery time were measured while exposing under 1% hydrogen, exhibiting the response and recovery time of ~30 min and ~15 min, respectively (Figure 3b). In addition, the effect of relative humidity (RH) on the sensor output was also measured. It can be noticed that the output voltage is obviously reduced when the RH is increased from 47% to 65%, negatively affecting triboelectric output voltage. Lastly, the sensor responses were calculated from multiple devices and plotted as a function of exposing H_2_ concentrations (Figure 4d). Here, the sensor responses were calculated using the equation *∆V/V_0_*, where *V_0_* is the output voltage measured while being exposed to dry air, *V_s_* is the output voltage under exposure to a specific H_2_ concentration, and *∆V* (= *V_s_* − *V_0_*) is a relative output voltage change between two states. A gradual increase of sensor response can be noticed as H_2_ concentration increases. We also note that the sensor response was more widely varied when the H_2_ concentration exceeds 2%. Using the obtained sensor responses, the sensitivity of the hydrogen sensor is subsequently extracted, where the sensitivity is calculated using the equation, $S=\frac{d\left(∆V/V_{0}\right)}{d\left(\left[H_{2}\right]\right)}$. Up to 1% H_2_ exposures, higher sensitivity, 0.75, was obtained. Above 2% H_2_ exposures, on the other hand, the sensitivity is reduced to 0.2, indicating the saturation of H_2_ molecules absorption in Pd lattice above this level.

## 3. Materials and Methods

### 3.1. Measurement and Characterization

Triboelectric output voltages were measured while exposing the device in different H_2_ gas concentrations. We maintained total flux of dry air and H_2_ as 200 sccm. For each H_2_ concentration, the sample was exposed to each H_2_ concentration for 30 min, which is sufficient reaction time for Pd in the presence of H_2_ gas. Different concentrations of H_2_ were introduced into the chamber by mixing with dry air at the specific ratios using mass flow control (MFC) units. For all measurement, a constant force of 0.1 MPa was applied using the custom-made pushing machine installed inside of a gas chamber at a frequency of 0.5 Hz and the distance between two electrodes was kept as 3.5 cm. The output voltages from the sensor were measured by connecting to the oscilloscope (Waverunner 2 LT 354, Lecroy, Chestnut Ridge, NY, USA). The Pd-coated layer was characterized by using both TEM (JEM-ARM200F, JEOL, Tokyo, Japan) and EDS (HD-2300A, Hitachi, Tokyo, Japan) before gas sensing measurement.

### 3.2. Fabrication of Triboelectric H_2_ Gas Sensor

For fabrication of triboelectric H_2_ gas sensors, the surface of ITO (130 nm)-coated PET (130 μm) film (Sigma Aldrich, Darmstadt, Germany) was first cleaned with IPA, acetone, and DI water, respectively, followed by N_2_ blow dry. After cleaning the film, Pd (10 nm) was thermally evaporated on an ITO surface of the film. Next, the counter contact surface, consisting of PET/ITO film, was prepared. Here, Pd-coated ITO surface was contact-electrified with the PET side of the counter contact surface. Electrical connection between ITO electrodes of the two friction surfaces was made using copper wires. For electrical connection of the ITO surface and copper line as an electrode, the electrode was soldered by soldering with silver paste. Each prepared surface was then mounted on the custom-made pushing machine installed inside a gas test chamber.

## 4. Conclusions

In summary, we demonstrated self-powered H_2_ gas sensor utilizing triboelectric effects. Due to WF change of Pd-coated surface in the presence of H_2_, triboelectric output voltage of the sensor was varied proportionally. Using this mechanism, different concentrations of H_2_ were detected during contact electrification between two friction surfaces without using an external battery. Specifically, the output voltage of H_2_ sensor was increased in proportion to H_2_ concentration. The WF of PdH*_x_* moves close to a donor state of PET, and the charge transfer between the two friction surfaces can be facilitated. Triboelectric H_2_ sensor demonstrated reproducible and sensitive responses up to 1% H_2_. The approach introduced here can be easily adopted to development of triboelectric gas sensors detecting other gas species and other self-powered electronics devices.

## Abbreviations

The following abbreviations are used in this manuscript:

H_2_	Hydrogen
Pd	Palladium
PdH*_x_*	Palladium Hydride
IPA	Isopropyl Alcohol
DI water	Deionized Water
ITO	Indium Tin Oxide
PET	Polyethylene Terephthalate
TEM	Transmission Electron Microscopy
EDS	Energy-Dispersive X-ray Spectroscopy
MFC	Mass Flow Control
WF	Work Function
